# Extracellular superoxide production is a widespread photoacclimation strategy in phytoplankton

**DOI:** 10.1093/ismeco/ycaf179

**Published:** 2025-11-27

**Authors:** Sydney Plummer, Susan Garcia, Julia M Diaz

**Affiliations:** Geosciences Research Division, Scripps Institution of Oceanography, University of California San Diego, La Jolla, CA 92093, USA; Department of Biology, The University of Alabama at Birmingham, Birmingham, AL 35294, USA; Geosciences Research Division, Scripps Institution of Oceanography, University of California San Diego, La Jolla, CA 92093, USA; School of Oceanography, University of Washington, Seattle, WA 98195, USA; Geosciences Research Division, Scripps Institution of Oceanography, University of California San Diego, La Jolla, CA 92093, USA

**Keywords:** reactive oxygen species, flavoenzymes, Prochlorococcus, photophysiology, superoxide, phytoplankton, light stress, photosynthesis-irradiance, photoacclimation, photoprotection

## Abstract

Phytoplankton help control the habitability of Earth by serving as the base of marine food webs, producing approximately half of the planet’s oxygen, and sequestering carbon dioxide from the atmosphere. As global changes accelerate through the Anthropocene, phytoplankton communities face multiple stressors, such as shifting patterns in ocean circulation, and associated changes in light exposure. The health of the oceans depends on phytoplankton responses to these stressors; however, the physiological processes involved in light stress are not fully understood. Here, we surveyed 16 representative phytoplankton and show that most produce extracellular superoxide, an otherwise damaging reactive oxygen species, as a widespread strategy to acclimate to light stress. Indeed, all species adjusted extracellular superoxide production as a function of light exposure, which was modeled with a modified photosynthesis-irradiance (PI) curve. Furthermore, the flavoenzyme inhibitor diphenyl iodonium (DPI) quenched extracellular superoxide production and led to declines in viability and photosynthetic health in 13 out of 16 species. The negative effect of DPI on photosynthetic health was stronger with increasing light, consistent with inhibition of a photoprotective process. Taken together, these results support the hypothesis that phytoplankton mitigate light stress through enzyme-mediated production of extracellular superoxide. These results imply that daytime rates of biological superoxide production in the marine environment are substantially underestimated by dark measurements. Furthermore, phytoplankton photoacclimation may alter superoxide production rates in future oceans impacted by changes in water column structure and light exposure.

## Introduction

Reactive oxygen species (ROS) are prevalent throughout aquatic environments like the oceans, where they contribute to biogeochemical cycling. ROS are transient intermediates in the reduction of oxygen to water that arise via biological and nonbiological pathways, including photochemical production [1]. The reactive nature of ROS such as hydrogen peroxide (H_2_O_2_) and superoxide (O_2_^−^) has been linked to the transformation of important elements in aquatic systems. Indeed, superoxide and subsequent ROS decrease available oxygen [2] and alter the speciation of harmful (e.g. Hg) [3] and nutrient metals (e.g. Fe, Cu) [4, 5]. ROS also contribute to carbon remineralization [1, 6, 7], especially through the action of hydroxyl radicals that can arise from superoxide [8]. In these ways, ROS influence ecosystem health and productivity.

Aerobic metabolism inevitably leads to the production of ROS, and ROS are thus generated by many organisms. Phytoplankton and bacteria are recognized as major sources of extracellular ROS (eROS) in the marine environment [9] that can rival abiotic (i.e. photochemical) contributions [5, 10]. Production rates of eROS by microbes are dynamically regulated via membrane-associated enzymes and can shift as a function of the environment (e.g. cell density, light levels) as well as physiology (e.g. size, growth stage) [8, 11, 12]. Biological ROS are often regarded as harmful byproducts of metabolism. Indeed, ROS, especially the hydroxyl radical, react with and damage biological molecules [9], and antioxidants help protect against such oxidative stress. Yet ROS production can also benefit marine biota [8, 13–15]. For example, there is evidence that ROS can help macroalgae defend against both pathogens [13] and grazers [14, 15]. In phytoplankton, production of eROS may play central roles in physiology, such as growth regulation and signaling, as well as acclimation to external stimuli [8], including excess light [16].

Excess light can cause reductive stress, which is characterized by the accumulation of reducing equivalents, such as NADPH, relative to their oxidized forms, such as NADP^+^. In phototrophs, the ratio of NADPH:NADP^+^ increases with increasing light levels [17]. Yet there can be a relative “excess” of NADPH, even at optimal light levels, because the ratio of ATP:NADPH produced during photosynthesis (~1.3) is estimated to be lower than what is needed to fuel carbon fixation (1.5) [18, 19]. In response to reductive stress, phytoplankton commonly employ energy-dissipating strategies across a wide range of light levels, such as nonphotochemical quenching [20], cyclic electron flow [21], and the Mehler reaction [22]. Enzymes (e.g. NADPH oxidases) that oxidize NADPH to generate eROS [23] could be another mechanism to deal with and prevent reductive stress.

We recently reported that the diatom *Thalassiosira oceanica* uses a transmembrane flavoenzyme similar to NADPH oxidases to transfer electrons from cellular NADPH to dissolved oxygen on the outside of the cell, thereby making extracellular superoxide (${\text e}{\text O_2^-}$). Furthermore, we confirmed that this process is dynamically controlled as a function of light exposure and helps prevent reductive stress under high irradiance [16]. However, it is unknown whether the ${\text e}{\text O_2^-}$-driven photoacclimation response observed in *T. oceanica* is widespread across phytoplankton taxa. If this process is conserved, it would have implications for biological rates of ${\text e}{\text O_2^-}$ production in the ocean, coupled biogeochemical cycles, and marine ecosystem functioning. Therefore, we surveyed a wide diversity of phytoplankton, including representatives of cyanobacteria and all major eukaryotic lineages, to determine how prevalent the photoprotective role of ${\text e}{\text O_2^-}$ production may be. We show that all the tested phytoplankton adjust ${\text e}{\text O_2^-}$ production as a function of light exposure and that most of them use it as a beneficial strategy to photoacclimate. Because this photoacclimation strategy is widespread, current measurements of microbial ${\text e}{\text O_2^-}$ production in the ocean, which are largely determined in the absence of light [2], are likely underestimated. Furthermore, we suggest that future, climate-driven shifts in surface ocean habitats may alter biological rates of ${\text e}{\text O_2^-}$ production.

## Materials and Methods

### Phytoplankton strains

All phytoplankton strains were obtained from the National Center for Marine Algae and Microbiota (NCMA) at Bigelow Laboratory for Ocean Sciences, except for the following strains: *Karenia brevis* ARC5 from the Algal Resources Collection at the University of North Carolina Wilmington (www.algalresourcescollection.com), *Ostreococcus tauri* OTH95 from the Palenik lab (Scripps Institution of Oceanography), *Dunaliella* sp. 15-1a from the Bowman lab (Scripps Institution of Oceanography), *Phaeodactylum tricornutum* CCAP 1055/1 from the Allen lab (J. Craig Venter Institute), and *Prochlorococcus marinus* NATL2A and MIT9312 from the Chisholm lab (Massachusetts Institute of Technology).

### Phytoplankton cultivation and growth tracking

Phytoplankton were grown in 0.2 μm filtered natural seawater that was amended with nutrients prior to being autoclaved (121°C, 20–40 min). Media types included SN [24], L1 with the addition of silicic acid [25], f/2, f/2 with the addition of silicic acid [26], or Pro99 [27] (Supplementary Table 1). All experimental cultures were begun with stationary phase inoculum except for *P. marinus* strains, which were begun with exponential phase inoculum. Cultures were maintained in borosilicate culture tubes with caps at 18°C or 23°C under cool, white light (14:10 light dark cycle) (Supplementary Table 1).

Growth was monitored by observing *in vivo* chlorophyll fluorescence using a handheld *Aqua*fluor® fluorometer (Turner Designs, San Jose, CA, USA) or cell abundance (cells ml^−1^) using a Guava® easyCyte flow cytometer (Luminex, Austin, TX, USA). Flow cytometry samples were analyzed by running live samples in a 96-well plate at a low flow rate of 0.24 μl s^−1^ for 3 min or until at least 1000 particles were counted. Instrument performance was validated daily using instrument specific beads. For analyses, gates of cell populations were created based on diagnostic red fluorescence and forward scatter signals of healthy, exponentially growing cells (exemplified by Supplementary Fig. 1). Specific growth rate (d^−1^) was calculated by finding the slope of the regression of the natural log-normalized cell abundance or fluorescence over time during exponential phase. Exponential phase was defined as the natural log-linear (R^2^ ≥ 0.98) portion of cell abundance or fluorescence over time. All experiments were conducted on exponentially growing cells, unless otherwise stated.

### ${\text e}{\text O_2^-}$ measurements

Production of ${\text e}{\text O_2^-}$ by phytoplankton cells was measured using the flow-through FeLume (II) analytical system (Waterville Analytical, Waterville, Maine, USA) via reaction with the ${\text O_2^-}$-specific chemiluminescent probe methyl *Cypridina* luciferin analog (MCLA), as previously described [11, 28, 29]. To do so, cells were gently deposited onto an inline filter (0.22 μm for most strains, except 0.1 μm for cyanobacteria and smaller picoeukaryotes, polyethersulfone membrane, 13 mm diameter) using a syringe and continuously washed (2 ml min^−1^) with phosphate-buffered artificial seawater (20 mM phosphate; pH = 7.6) that matched the salinity of the culture media. The ${\text e}{\text O_2^-}$ within this cell-free effluent reacted with the MCLA reagent [4 μM MCLA, 0.1 M MES, 75 μM diethylenetriamine pentaacetic acid (DTPA); pH = 6] at the center of a spiral flow cell sitting below a photomultiplier tube housed within a light-tight box. Chemiluminescent data emitted from the reaction between ${\text e}{\text O_2^-}$ and MCLA were collected in real time using Waterville Analytical software. Autooxidation of the MCLA reagent generates O_2_^−^. Therefore, chemiluminescence was measured from cell-free blanks (i.e. MCLA reagent + artificial seawater) just prior to depositing phytoplankton cells onto the inline syringe filter for each analysis. These blank chemiluminescent signals were subtracted from the subsequent biological chemiluminescent signals (i.e. MCLA reagent + artificial seawater + cells). A steady-state signal was obtained by allowing chemiluminescent signals of both blanks and cells to stabilize ($\le$5% CV) for at least 1 min. Superoxide dismutase (SOD; final concentration of ~800 U L^−1^) was added at the end of each analysis to confirm chemiluminescent signals were due to O_2_^−^.

FeLume calibrations were performed with standard additions of potassium superoxide (KO_2_), as previously described [11, 29]. First, primary KO_2_ stocks were prepared by dissolving in a basic solution (0.03 N NaOH, pH = 12.5; 90 μm DTPA). Then, absorbance of primary KO_2_ stocks [30] was measured at 240 nm before immediately being added to artificial seawater. The decay of O_2_^−^ was measured in this solution on the FeLume. The primary KO_2_ stocks were again measured at an absorbance of 240 nm after addition of SOD (~ 800 U L^−1^, final concentration). Absorbance measurements of KO_2_ primary stocks before and after SOD addition were used to quantify the O_2_^−^ concentrations by applying the extinction coefficient of O_2_^−^ corrected for production of H_2_O_2_ at 240 nm and a pH of 12.5 (2183 L mol^−1^ cm^−1^). Biological chemiluminescent signals were converted to steady-state ${\text e}{\text O_2^-}$ concentrations by dividing the chemiluminescent signals by the sensitivity of the analysis (chemiluminescent counts pM^−1^) obtained through standard addition calibrations. Then ${\text e}{\text O_2^-}$ concentrations (pM) were converted to production rates by multiplying by the flow rate (1 or 2 ml min^−1^), dividing that number by the number of cells loaded on the inline syringe filter, and converting final units to amol cell^−1^ h^−1^ or amol μm^−2^ h^−1^ to normalize to cell surface area. To ensure the same number of healthy cells was analyzed between replicates during an experiment, a cell concentration was first obtained via flow cytometry as detailed above. These production rates account for simultaneous production and decay of ${\text e}{\text O_2^-}$ and are therefore net production rates.

### Culture experiments

Four different types of experiments were performed on all 16 phytoplankton strains: one growth experiment, two ${\text e}{\text O_2^-}$ production experiments, and one photophsysiological experiment. Due to time and volume constraints, each strain was cultivated separately in 25 ml volumes for each experiment.

### Growth experiment

In the growth experiment, cultures were grown with the addition of the flavoenzyme inhibitor diphenyl iodonium (DPI). DPI irreversibly binds to flavin adenine dinucleotide (FAD)/ flavin mononucleotide (FMN) and consequently blocks electron transfer activity [31]. Treatments included DPI (2 μM and 0.02 μM final concentrations) dissolved in 10% DMSO, 10% DMSO (0.03% v/v final concentration), or no treatment (unamended). The DPI and DMSO treatments (75 μl each) were added one time after the beginning of exponential growth phase. In these experiments, growth was monitored by obtaining daily fluorescence (*P. marinus* strains) or cell abundance (all other strains). All experiments were conducted at growth irradiance (Supplementary Table 1), except a control experiment with *T. oceanica*, which tested the effect of DPI (2 μM dissolved in 10% DMSO; and 10% DMSO, 0.03% v/v final concentration) at 10% growth irradiance.

### Production of ${\text e}{\text O_2^-}$ experiments

In the first type of ${\text e}{\text O_2^-}$ production experiment, production of ${\text e}{\text O_2^-}$ by phytoplankton cells was measured in the presence of DPI or the solvent control, DMSO. To do so, the artificial seawater (20 mM phosphate; pH = 7.6) that continuously washes cells during analysis was amended daily by heating and stirring for 1 h, adding 20 μM DPI dissolved in 0.3% DMSO, or adding 0.3% DMSO alone (final concentrations), and allowing the solution to cool to room temperature. For these experiments, ${\text e}{\text O_2^-}$ measurements were collected at the growth irradiance of each phytoplankton strain (Supplementary Table 1). Irradiance was emitted from a dimmable soft white LED light bulb controlled with a manual dimmer and placed directly above the sample. A standard light bulb, which emits light in the visible range, and little ultraviolet irradiation compared to natural sunlight [32], was used rather than a full spectrum light source to minimize abiotic ROS production while stimulating photosynthesis [9]. Irradiance was monitored with a micro quantum photosynthetically active radiation (PAR) sensor (Walz, Effeltrich, Germany). Data analysis and calculation of ${\text e}{\text O_2^-}$ production rates in the presence of DPI followed that of Plummer *et al.* [11] and is detailed above.

In the second type of ${\text e}{\text O_2^-}$ production experiment, ${\text e}{\text O_2^-}$ production was measured as a function of increasing irradiance from ambient light levels (3–6 μmol m^−2^ s^−1^) to ~2250 μmol m^−2^ s^−1^ using a dimmable soft white LED light bulb, as previously described [16]. Then, these ${\text e}{\text O_2^-}$ production rates (${P}^{O_2^{-}}$) and irradiance (*E*) data were fit to an equation modified for ${\text e}{\text O_2^-}$ production from the double exponential photosynthesis-irradiance (PI) model by Platt *et al.* [33] following the methods of Diaz *et al.* [16]:


(1) \begin{equation*} {P}^{O_2^{-}}={P}_D^{O_2^{-}}+{P}_S^{O_2^{-}}\left[1-{e}^{\frac{-\alpha }{P_S^{O_2^{-}}}E}\right]{e}^{\frac{-\beta }{P_S^{O_2^{-}}}E} \end{equation*}

Where the fitted constants included *α*, the initial linear slope of the best fit curve; *β*, the parameter describing the decrease of ${\text e}{\text O_2^-}$ at high irradiances; ${P}_D^{O_2^{-}}$, the net production rate of ${\text e}{\text O_2^-}$ in the dark; and ${P}_S^{O_2^{-}}$, an estimate of the light-saturated rate of net ${\text e}{\text O_2^-}$ production if *β* = 0. Light-saturated rates of net ${\text e}{\text O_2^-}$production (${P}_M^{O_2^{-}}$) and the minimum saturating irradiance of net ${\text e}{\text O_2^-}$ production (${E}_k^{O_2^{-}}$) were calculated by the following equations:


(2) \begin{equation*} {P}_M^{O_2^{-}}={P}_S^{O_2^{-}}\left(\frac{\alpha }{\alpha +\beta}\right){\left(\frac{\beta }{\alpha +\beta}\right)}^{\beta /\alpha }+{P}_D^{O_2^{-}} \end{equation*}

(3) \begin{equation*} {E}_k^{O_2^{-}}=\frac{P_M^{O_2^{-}}}{\alpha } \end{equation*}

Taxon-specific constants are reported in Supplementary Table 2.

### Photophysiology experiment

In the photophysiology experiment, photophysiology was monitored using either a Satlantic fluorescence induction and relaxation (FIRe) fluorometer system (Sea-bird Scientific, Bellevue, Washington, USA) or a pulse amplitude modulation (WATER-PAM) fluorometer (Walz, Effeltrich, Germany) similar to Diaz *et al.* [16]. Prior to measurements, phytoplankton were incubated for ~30 min with 2 μM DPI dissolved in 0.2% DMSO or 0.2% DMSO (final concentrations), either at their growth irradiance (Supplementary Table 1) for light-acclimated samples, or at dark conditions for dark-acclimated samples. Photochemical efficiency of photosystem II (PSII) was determined by calculating photosynthetic efficiency (F_v_/F_m_) in the dark-acclimated or light-acclimated state using the equation:


(4) \begin{equation*} {F}_v/{F}_m=\frac{F_m-{F}_o}{F_m} \end{equation*}

where ${F}_m$ is the maximum fluorescence yield, and ${F}_o$ is the minimum fluorescence yield. Decreases in F_v_/F_m_ correspond to increases in photoinhibition [34, 35].

### Rate calculations of ${\text e}{\text O_2^-}$ production in the North Pacific subtropical gyre

We used our culture results to determine dark and light-driven rates of biological ${\text e}{\text O_2^-}$ production in the North Pacific Subtropical Gyre (NPSG). First, we calculated *in situ* irradiance levels (${E}_z$; μmol m^−2^ s^−1^) using an equation that describes how light intensity decreases exponentially with depth:


(5) \begin{equation*} {E}_z={E}_0{e}^{- kz} \end{equation*}

Where ${E}_0$is defined as the irradiance level at the ocean surface obtained from seasonal PAR ranges in the NPSG [36] (324 to 613 μmol m^−2^ s^−1^ with a median of 481 μmol m^−2^ s^−1^), $k$ is the average light attenuation coefficient throughout the seasons (0.04 m^−1^) [36], and *z* is depth (m). We used the long-term Hawaii Ocean Time-series (HOT) station ALOHA as a representative site in the NPSG. Depth (*z*) was defined as the mixed layer depth (MLD) unless otherwise noted. MLD was determined from the publicly available HOT dataset [37] over all years and seasons at station ALOHA. We defined the fifth and 95th percentiles (27–109 m) as the range of typical MLD values, and we determined the median MLD to be 55 m (Supplementary Table 3). We assumed a MLD shallowing of 20 m in this region by the year 2200 based on the study by Luo and Rothstein [38], and therefore defined the median future MLD to be 35 m (Supplementary Table 4).

In situ biological ${\text e}{\text O_2^-}$ production rates (${P}_{in\ situ}^{O_2^{-}}$; nmol L^−1^ d^−1^) can be described as a function of *in situ* irradiance levels (*E_z_*, determined by equation 5) using a modified form of equation (1):


(6) \begin{equation*} {P}_{in\ situ}^{O_2^{-}}=A\times \left({P}_D^{O_2^{-}}+{P}_S^{O_2^{-}}\left[1-{e}^{\frac{-\alpha }{P_S^{O_2^{-}}}{E}_z}\right]{e}^{\frac{-\beta }{P_S^{O_2^{-}}}{E}_z}\right) \end{equation*}

where, $\alpha$, $\beta$, ${P}_D^{O_2^{-}},\mathrm{and}\ {P}_S^{O_2^{-}}$ are taxon-specific constants determined from equation 1 (Supplementary Table 2) and *A* is the taxon-specific cell abundance in the environment (cells L^−1^; Supplementary Table 3). For these calculations, we utilized data from oligotrophic (e.g. *Synechococcus* sp. WH8102 instead of WH5701; *T. oceanica* instead of *T. pseudonana*) and environmentally relevant representatives (calcifying *Emiliania huxleyi* CCMP 371 instead of noncalcifying CCMP 374). We assumed that cell abundances in the future would not change. Rates of dark biological ${\text e}{\text O_2^-}$ production were calculated using *E_z_* = 0.

### Statistical analyses

All statistical analyses were performed in JMP Pro statistical software (SAS Institute Inc.,) or Microsoft Excel. A paired Student’s t-test (two-sample, two-tailed) and an unpaired, Student’s t-test (two-sample, two-tailed) were used to determine the effect of DPI on ${\text e}{\text O_2^-}$ production rates and phytoplankton growth rates, respectively. A Tukey-Honest Significance Difference (HSD) test was used to determine the effect of DPI on efficiency of PSII in light- acclimated and dark-acclimated states. The significance threshold (alpha) was set to 0.05 for all statistical analyses.

## Results and Discussion

### Superoxide production varies widely among strains

**Figure 1:**
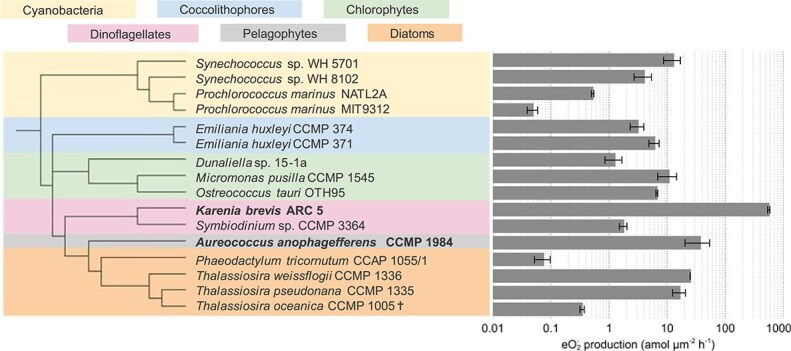
Surface area-normalized ${\text e}{\text O_2^-}$ production measurements were conducted under typical growth irradiance for each strain (see Supplementary Table 1). Leaf colors on phylogenetic tree indicate the different taxonomic groups. Bold font indicates harmful algal bloom-forming strains. Cell surface areas are shown in Supplementary Table 5. † indicates data from [16].

We measured ${\text e}{\text O_2^-}$ production rates from 16 strains of prokaryotic and eukaryotic phytoplankton spanning a diversity of taxonomic groups and ecotypes (Fig. 1; Supplementary Table 1). These phytoplankton included representatives of the numerically dominant cyanobacteria *Synechococcus* and *Prochlorococcus*; *E. huxleyi*—a widespread coccolithophore found from the equator to subpolar regions [39]; ecologically diverse green algae from the chlorophyte group; three species of *Thalassiosira*, one of the most abundant genera of diatoms [40]; and harmful bloom-forming algae from the dinoflagellate and pelagophyte groups. Since cell size influences ${\text e}{\text O_2^-}$ production rates [28, 41, 42], we normalized production rates to cell surface area (Supplementary Table 5). Production of ${\text e}{\text O_2^-}$ varied widely among the strains surveyed (Fig. 1 and Supplementary Fig. 2). For example, production rates of ${\text e}{\text O_2^-}$ measured under typical growth irradiances for each strain (see Supplementary Table 1) spanned 10^−3^–10^2^ amol μm^−2^ h^−1^; however, most phytoplankton strains produced ${\text e}{\text O_2^-}$ in the 10^−1^–10^1^ amol μm^−2^ h^−1^ range (Fig. 1 and Supplementary Fig. 2). Similar to previous results showing substantial ${\text e}{\text O_2^-}$ production by harmful algal species [43], the two highest ${\text e}{\text O_2^-}$ producers were the harmful bloom-forming algae *K. brevis* ARC 5 and *Aureococcus anophagefferens* CCMP 1984. While *A. anophagefferens* produced similar amounts of ${\text e}{\text O_2^-}$ to several nonharmful strains (10^1^ amol μm^−2^ h^−1^), *K. brevis* produced an order of magnitude more. *Prochlorococcus* (strain MIT9312) had the lowest rate of ${\text e}{\text O_2^-}$ production, consistent with previous observations [44]. Intraspecific variation was observed in *Prochlorococcus,* where *P. marinus* NATL2A produced 10-fold more ${\text e}{\text O_2^-}$ than *P. marinus* MIT9312. This finding may be related to the broader genomic capabilities of NATL2A, which contains more high-light induced photoprotective proteins than MIT9312 [45, 46].

### Irradiance drives superoxide production

**Figure 2:**
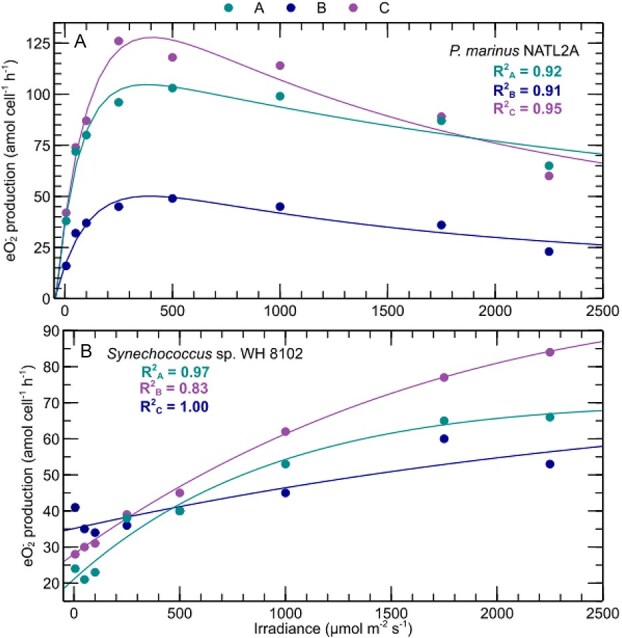
Irradiance versus ${\text e}{\text O_2^-}$ production. *P. Marinus* NATL2A (A) shows the typical photoinhibition response observed in 15 of 16 strains studied, and *Synechococcus* sp. WH 8102 (B) shows an atypical response lacking photoinhibition. Irradiance and ${\text e}{\text O_2^-}$ production rate data were fit with a modified photosynthesis-irradiance model [16] (lines). See Materials and Methods and Supplementary Table 2. Each color represents a different biological replicate. Additional strains are shown in Supplementary Fig. 3.

Production of ${\text e}{\text O_2^-}$ has been attributed to photosynthesis in previous studies, usually based on ${\text e}{\text O_2^-}$ production measurements conducted at a limited number or range of light levels [47–50]. To expand on these observations, we measured and modeled ${\text e}{\text O_2^-}$ production across a wide range of irradiances. In all phytoplankton tested, irradiance drove ${\text e}{\text O_2^-}$ production (Fig. 2 and Supplementary Fig. 3). Typically, ${\text e}{\text O_2^-}$ production followed a pattern similar to a photosynthesis-irradiance model [16] (Supplementary Table 2; avg. ± SD of R^2^ in all strains = 0.95 ± 0.05). Consistent with the expected light-driven control of photosynthetic rates, ${\text e}{\text O_2^-}$ production rates increased linearly at low irradiances, became saturated at intermediate irradiances, and decreased at highest irradiances via photoinhibition (Supplementary Fig. 3 and exemplified by *P. marinus* NATL2A in Fig. 2a). The single exception to this trend was observed in the oligotrophic *Synechococcus* strain (WH 8102), in which ${\text e}{\text O_2^-}$ production was not photoinhibited, yet still increased as a function of light (Fig. 2b). These findings support the hypothesis that photosynthetic processes control ${\text e}{\text O_2^-}$ in diverse phytoplankton species.

### Flavoenzymes mediate ${\text e}{\text O_2^-}$ production

Superoxide (pKa = 4.8) is an anion at typical cellular pH and has a short intracellular lifetime (~10^−5^ s) [12]. Therefore, ${\text e}{\text O_2^-}$ does not readily cross the plasma membrane that separates the cell from its surrounding environment and is unlikely to arise from passive diffusion or cell lysis [51]. Instead, biological ${\text e}{\text O_2^-}$ production is typically mediated by cell surface enzymes that catalyze electron transport across the plasma membrane [12, 51]. In phytoplankton, this transplasma membrane electron transport is thought to be driven by flavoenzymes that oxidize the cellular reductant NADPH, including NADPH oxidase (NOX) [16, 43, 49, 52–54] and glutathione reductase [16, 55], which are inhibited by DPI [31, 55].

**Figure 3:**
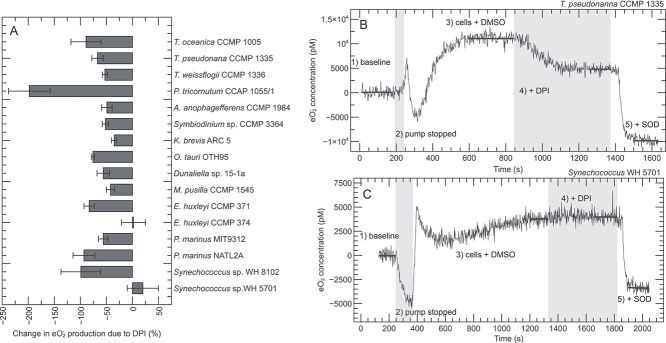
${\text e}{\text O_2^-}$ production in the presence or absence of the flavoenzyme inhibitor DPI. Percent change in ${\text e}{\text O_2^-}$ production due to application of DPI (A). Error bars show standard deviation of the mean (*n* = 3 biological replicates). Time series of biological ${\text e}{\text O_2^-}$ production showing the typical inhibiting effect of DPI observed in 14 of 16 strains and exemplified by *T. pseudonanna* CCMP 1335 (B), and the atypical result showing no effect of DPI exemplified by *Synechococcus* sp. WH 5701 (C). Traces are split into five regions of analysis, as indicated (see Materials and Methods). Horizontal bars show stable signals from which the production rates were calculated. DPI was dissolved in DMSO, which had no impact on ${\text e}{\text O_2^-}$ production, as expected [16]. Negative ${\text e}{\text O_2^-}$ concentrations in the presence of superoxide dismutase (SOD) and changes in ${\text e}{\text O_2^-}$ production that are <−100% reflect degradation of background ${\text e}{\text O_2^-}$ originating from the analytical reagent (see Materials and Methods). Data for *T. oceanica* CCMP 1005 are from Diaz *et al.* [16].

Here, we applied DPI and found that it inhibited ${\text e}{\text O_2^-}$ production in 14 of the 16 phytoplankton strains examined (Fig. 3a and Supplementary Fig. 2), consistent with a predominant role for flavoenzymes. Indeed, DPI quenched ${\text e}{\text O_2^-}$ production on a timescale of minutes in many strains (exemplified by *T. pseudonanna* CCMP 1335 in Fig. 3b). Yet here and in other studies [16, 49, 52–54], DPI did not completely eliminate ${\text e}{\text O_2^-}$ production in most organisms (Fig. 3a and Supplementary Fig. 2), suggesting that enzymes in addition to the flavoenzyme group are involved in ${\text e}{\text O_2^-}$ production. In the coccolithophore *E. huxleyi* CCMP 374 and the coastal *Synechococcus* strain WH 5701, ${\text e}{\text O_2^-}$ production was insensitive to DPI, indicating that flavoenzymes may not produce ${\text e}{\text O_2^-}$ in these organisms (Fig. 3 and Supplementary Fig. 2).

### Flavoenzyme/${\text e}{\text O_2^-}$ inhibitor impairs photosynthetic health and viability

**Figure 4:**
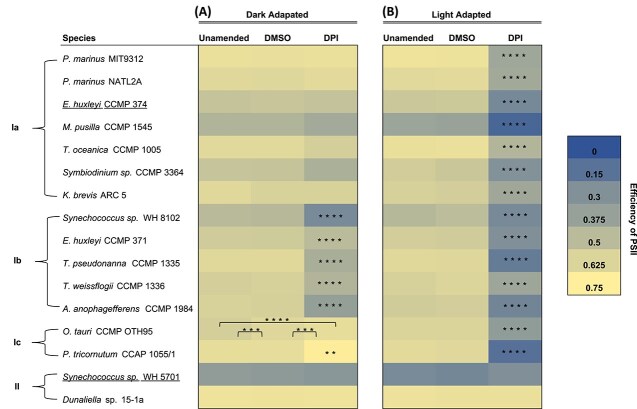
Efficiency of PSII in the presence or absence of the flavoenzyme inhibitor DPI. Phytoplankton were acclimated to (A) their growth irradiance (light-acclimated) or (B) darkness (dark-acclimated) and chemical treatments for ~30 min prior to measurements. All measurements were conducted in 0.2% DMSO, except the unamended control. Significant differences of the mean F_v_/F_m_ (*n =* 3 biological replicates) between treatments were found using a Tukey-Honest Significance Difference (HSD) test. P-values are indicated by asterisks, where ^**^, ^***^, and ^****^ signifies a *P*-value of < .01, < .001, and < .0001, respectively. Responses of efficiency of PSII to DPI are categorized into four groups: Ia, inhibition under light adaptation only; Ib, inhibition under both light and dark adaptation; Ic, inhibition under light adaptation, yet stimulation under dark conditions; and II, no effect. Strains that are underlined did not show DPI-inhibitable ${\text e}{\text O_2^-}$ production. Data for *T. oceanica* CCMP 1005 are from Diaz *et al.* [16].

Given the confirmed photoprotective role of ${\text e}{\text O_2^-}$ production in *T. oceanica* [16], we hypothesized that DPI would impair photosynthetic health in most phytoplankton that exhibited DPI-inhibitable ${\text e}{\text O_2^-}$ production. Therefore, we examined the effect of DPI on the efficiency of photosystem II (F_v_/F_m_) under light and dark conditions on a timescale of tens of minutes (see Materials and Methods). F_v_/F_m_ represents the fraction of total absorbed light energy that is used in photosynthesis, with higher levels typically representing better photosynthetic health. We found that DPI inhibited F_v_/F_m_ in 14 of 16 strains and that this inhibition was dependent on the presence of light (Fig. 4. groups Ia-c). In the majority of strains, DPI decreased F_v_/F_m_ under light-acclimated conditions but not dark-acclimated conditions (group Ia), while in other strains, DPI decreased F_v_/F_m_ under both light and dark conditions (group Ib). In two strains, DPI inhibited F_v_/F_m_ under light conditions yet stimulated F_v_/F_m_ under dark conditions (group Ic). Overall, DPI inhibited ${\text e}{\text O_2^-}$ production and F_v_/F_m_ in the majority (13/16) of strains (Fig. 4), consistent with the hypothesized role for ${\text e}{\text O_2^-}$ production in supporting phytoplankton photophysiology.

**Figure 5:**
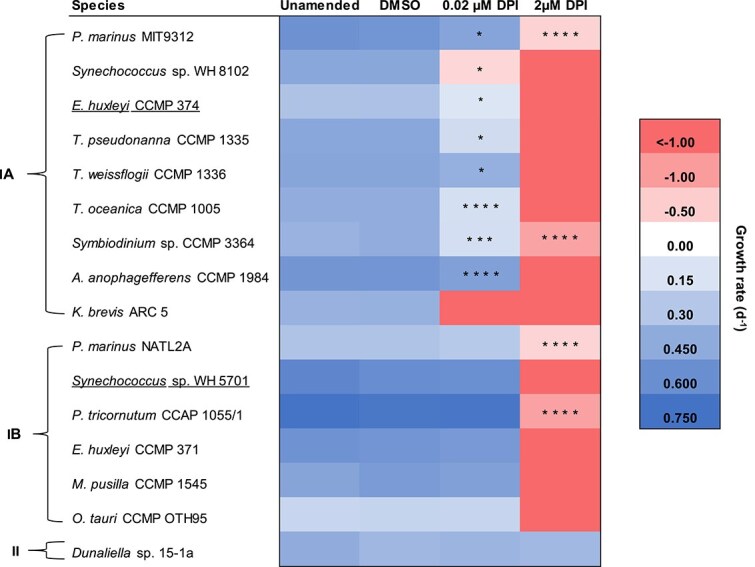
Specific growth rates in the presence or absence of the flavoenzyme inhibitor DPI. All cultures were grown in 0.03% DMSO, except the unamended control. Negative growth rates indicate culture death. Growth rates <−1.00 d^−1^ indicate that cells died at a rate that was faster than our limit of detection. Asterisks indicate significant differences of mean growth rates > −1.00 (*n =* 3 biological replicates) between the DPI and DMSO treatments using a Student’s t-test (unpaired, two-sample). Statistical results between all other conditions are shown in Supplementary Fig. 5. *P*-values are indicated by asterisks, where ^*^, ^**^, ^***^, and ^****^ signifies a *P*-value of < .05, < .01, < .001, and < .0001, respectively. Responses of growth rates to DPI are categorized into 3 groups: Ia, significant declines in both the 0.02 μM DPI and 2 μM DPI treatments; Ib, significant declines in only the 2 μM DPI treatment; and II, no effect. Strains that are underlined did not show DPI-inhibitable ${\text e}{\text O_2^-}$ production.

Based on the negative effect of DPI on ${\text e}{\text O_2^-}$ production and photosynthetic health in the majority of phytoplankton tested, we hypothesized that DPI would also impair viability. To test this hypothesis, we added two concentrations of DPI to early exponential phase cultures and compared their growth to DMSO and unamended controls (Supplementary Fig. 4). DPI inhibited growth and ultimately led to cell death in 15 out of 16 strains (Fig. 5 and Supplementary Fig. 5). Further, the effect of DPI was concentration-dependent in the majority of strains (group Ia), though some were only sensitive to the higher concentration of DPI (group Ib). Overall, DPI inhibited both ${\text e}{\text O_2^-}$ production and growth in the majority (14/16) of strains (Fig. 5), consistent with the hypothesized role for ${\text e}{\text O_2^-}$ production in promoting phytoplankton viability.

### Extracellular superoxide production supports phytoplankton health by aiding redox balance

In summary, for the 14 organisms showing DPI-inhibitable ${\text e}{\text O_2^-}$ production, DPI treatment also decreased photosynthetic efficiency and growth, eventually leading to cell death in all but one case (Table 1). Combined with the tight regulation of ${\text e}{\text O_2^-}$ production by photosynthesis, these findings are consistent with the hypothesis that ${\text e}{\text O_2^-}$ production supports the health of most phytoplankton by protecting against photodamage. It is unlikely that our findings could be explained by a broad and nonspecific cytotoxicity of DPI because adverse health outcomes were not always linked to the inhibition of ${\text e}{\text O_2^-}$ production by DPI and vice versa, such as in *Dunaliella* sp. 15-1a, *Synechococcus* sp. WH 5701, and *E. huxleyi* CCMP 374 (Table 1). Also, our photophysiological experiments show that for most strains, DPI was only toxic to photosynthetic health in the light and not in the dark (e.g. Fig. 4 groups 1a and 1c). Similarly, the negative effect of DPI on growth was dependent on irradiance, with DPI causing faster mortality of the diatom *T. oceanica* at full growth irradiance (within 24 h) than at ~10% growth irradiance (after 24 h and before 48 h) (Supplementary Fig. 6.). These light-dependent effects of DPI are consistent with the proposed light-dependent ${\text e}{\text O_2^-}$ photoprotection strategy driven by flavoenzymes.

**Table 1:** The effect of DPI on ${\text e}{\text O_2^-}$ production, growth, and photosynthetic efficiency (F_v_/F_m_) of phytoplankton studied.

Phytoplankton strain	Superoxide production	Growth	Photosynthetic efficiency
*Synechococcus* sp. WH 8102	-	-	-
*Synechococcus* sp. WH 5701	+/−	-	+/−
*P. marinus* MIT9312	-	-	-
*P. marinus* NATL2A	-	-	-
*E. huxleyi* CCMP 374	+/−	-	-
*E. huxleyi* CCMP 371	-	-	-
*O. tauri* OTH95	-	-	-
*Micromonas pusilla* CCMP 1545	-	-	-
*Dunaliella sp.* 15-1a	-	+/−	+/−
*Symbiodinium* sp. CCMP 3364	-	-	-
***K. brevis* ARC 5**	-	-	-
*T. weissflogii* CCMP 1336	-	-	-
*T. pseudonanna* CCMP 1335	-	-	-
*T. oceanica* CCMP 1005	-^*^	-	-^*^
*P. tricornutum* CCAP 1055/1	-	-	-
***A. anophagefferens* CCMP 1984**	-	-	-

Bold font indicates a HAB forming species. Effects of DPI include significant inhibition (−) or no effect (+/−).

^*^results from Diaz *et al.* [16].

Exceptions to the general trend we observed included the extremophile *Dunaliella* sp. 15-1a, in which DPI inhibited ${\text e}{\text O_2^-}$ production to a similar degree as other phytoplankton (Fig. 3), but DPI did not affect photosynthetic health or growth (Fig. 4 and 5 and Table 1), demonstrating that ${\text e}{\text O_2^-}$ production may not serve a photoprotective mechanism in this species. We could not test the potential photoprotective role of ${\text e}{\text O_2^-}$ production in *Synechococcus* sp. WH 5701 or *E. huxleyi* CCMP 374 because ${\text e}{\text O_2^-}$ production by these organisms was insensitive to DPI (Fig. 3). However, we did observe some impairments to health from DPI treatments with these strains (Fig. 4 and 5), consistent with the diverse physiological roles of flavoenzymes [56]. For instance, in *E. huxleyi* CCMP 374, DPI inhibited growth and photosynthetic health, whereas DPI only inhibited growth in *Synechococcus* sp. WH 5701 (Table 1).

Similar to other photoprotective pathways (see Introduction), our results are consistent with ${\text e}{\text O_2^-}$ production helping prevent reductive stress by contributing to optimal cellular NADPH levels, promoting intracellular redox balance, and supporting health in many diverse phytoplankton. However, outside of photosynthesis, ${\text e}{\text O_2^-}$ production may play an even broader role in biological redox homeostasis. The majority of biotic ${\text e}{\text O_2^-}$ production rates in laboratory and field settings have been measured in the absence of light to eliminate abiotic O_2_^−^ production via photochemical reactions [28, 44, 57, 58]. Indeed, phytoplankton produce ${\text e}{\text O_2^-}$ under dark conditions, but at lower rates than in the light [11, 16, 49, 50] (Fig. 2 and Supplementary Fig. 3). In the dark, these microbes likely produce ${\text e}{\text O_2^-}$ using light-independent sources of reducing power, such as NADPH derived from the oxidative pentose phosphate pathway. In fact, Yuasa *et al.* reported that the harmful bloom-forming microalga *Chattonella antiqua* produces elevated levels of ${\text e}{\text O_2^-}$ to adjust imbalanced NADPH:NADP^+^ ratios influenced by the oxidative pentose phosphate pathway that occur under nitrogen and phosphorous limitation [59]. In these ways, ${\text e}{\text O_2^-}$ production appears to be a cellular redox acclimation strategy that is controlled by nutrient availability, as well as light exposure in phytoplankton.

### Implications for light-driven superoxide production in the marine environment

Environmental rates of biological superoxide production are not fully understood because most observations have been conducted only under dark conditions. Our results advance the current understanding of biological superoxide production in the marine environment by illuminating potential dynamics in the daytime. For example, we can extrapolate the ${\text e}{\text O_2^-}$-irradiance response of each phytoplankton strain to the environment by accounting for its *in situ* cell abundance and level of light exposure, including darkness (see Materials and Methods). The sum of these taxon-specific rates represents ${\text e}{\text O_2^-}$ production by the phytoplankton community.

We applied the above approach to Station ALOHA, a long-term time series site in the North Pacific Subtropical Gyre. First, we estimated the phytoplankton-driven ${\text e}{\text O_2^-}$ production under dark conditions and compared our result to the only available measurements of biological ${\text e}{\text O_2^-}$ production at Station ALOHA, which were conducted in the dark [57]. Because half of our cultures including *Prochlorococcus* were axenic during our experiments (Supplementary Table 1), our calculation underestimates ${\text e}{\text O_2^-}$ degradation by heterotrophic bacteria, which would be significant in the environment [1]. Yet, our approach is conservative in the sense that we do not account for the influence of nutrient stress, which is likely prevalent in the oligotrophic NPSG, and would probably increase ${\text e}{\text O_2^-}$ production rates [59]. Nevertheless, our extrapolation of 9 nM d^−1^ at 0 μmol m^−2^ s^−1^ by the phytoplankton community agrees well with measurements by Roe *et al.* [57], who reported dark rates of ${\text e}{\text O_2^-}$ production at Station ALOHA that averaged between ~2.6 and 6 nM d^−1^. Due to the numerical dominance of cyanobacteria in the phytoplankton community at Station ALOHA (Supplementary Table 3 and references therein), *Prochlorococcus* and *Synechococcus* were the dominant contributors to ${\text e}{\text O_2^-}$ production under dark conditions, producing 87% and 11% of ${\text e}{\text O_2^-}$, respectively.

Next, we used a similar approach to determine the effect of light exposure on phytoplankton-driven ${\text e}{\text O_2^-}$ production rates at Station ALOHA. To do so, we used ocean surface measurements of photosynthetically active radiation [36] to calculate *in situ* PAR fluxes throughout the mixed layer [37]. *Prochlorococcus* and *Synechococcus* generated 82%–99% and 1–17% of phytoplankton-derived ${\text e}{\text O_2^-}$ across the range of light levels found throughout the mixed layer, respectively (see Materials and Methods). Additionally, based on data from Malmstrom *et al.* [60], we determined that the high-light adapted *Prochlorococcus* ecotypes (represented by MIT9312) constitute ~99–100% of the *Prochlorococcus* community within the mixed layer PAR range. Therefore, our subsequent analyses focused on *Prochlorococcus* MIT9312 based on its dominance. At the median annual PAR level and MLD, we estimate light-driven ${\text e}{\text O_2^-}$ production by *Prochlorococcus* to be 14 ± 7 nM d^−1^ (Supplementary Table 4), representing a ~50% increase over the modeled community ${\text e}{\text O_2^-}$ production rate under dark conditions. Our light-driven estimates of biological ${\text e}{\text O_2^-}$ production do not account for UV irradiance (see Materials and Methods), which has the potential to both diminish or amplify environmental O_2_^−^ production through its inhibitory effect on photosynthesis [61, 62], or through the abiotic photooxidation of organic matter [1], respectively. Nevertheless, our results indicate that PAR exposure has the potential to increase environmental rates of biological ${\text e}{\text O_2^-}$ production.

Light-driven ${\text e}{\text O_2^-}$ production by phytoplankton may have implications for future oceans impacted by climate change. For example, MLDs in the NPSG are predicted to shallow up to 20 m by the year 2200 [38]. We estimate that a 20 m shallowing of the historic median annual MLD at Station ALOHA would increase light-dependent ${\text e}{\text O_2^-}$ production to 21 ± 10 nM d^−1^ using the median annual PAR level (see Materials and Methods; Supplementary Table 4). This change corresponds to a 50% increase in ${\text e}{\text O_2^-}$ production compared to the historic median annual MLD (Supplementary Fig. 7). This estimate is likely conservative, because we did not account for potential climate-driven changes in *Prochlorococcus* abundances, which are predicted to increase in this region [63]. Elsewhere in the global ocean, enhanced water column stratification has contributed to the deepening of ocean MLDs worldwide [64]. And in polar regions, loss of sea ice has increased water column light exposure [65]. Phytoplankton photoacclimation is also predominant on a global scale [66]. Therefore, future work should consider how phytoplankton photoacclimation may alter environmental ${\text e}{\text O_2^-}$ levels, and the coupled cycling of oxygen, carbon, and metals, in response to climate-driven changes in water column structure and light exposure.

## Supplementary Material

Survey_Paper_Supplementary_Information_edits_3_ycaf179

Supplementary_Table_6_ycaf179

## Data Availability

The datasets generated during and/or analyzed during the current study are available from the corresponding authors on reasonable request.
