# Supplementary material for: Extracellular superoxide production is a widespread photoacclimation strategy in phytoplankton
Source: ISME Commun. 2025 Nov 27;5(1):ycaf179. doi: 10.1093/ismeco/ycaf179 (PMC12684733; doi:10.1093/ismeco/ycaf179)
Supplement: Survey_Paper_Supplementary_Information_edits_3_ycaf179 [file survey_paper_supplementary_information_edits_3_ycaf179.pdf]

Supplementary Information for

Extracellular superoxide production is a widespread photoacclimation strategy in phytoplankton

Sydney Plummer[1, †*], Susan Garcia[1,†], Julia M. Diaz[1*]

Affiliations

[1]Geosciences Research Division, Scripps Institution of Oceanography, University of California San Diego, La Jolla, CA 92093, USA

[†]Present Address:

Sydney Plummer, Department of Biology, The University of Alabama at Birmingham, Birmingham, AL 35294, USA

Susan Garcia, School of Oceanography, University of Washington, Seattle, WA 98195, USA

*Corresponding authors: plummers@uab.edu, j2diaz@ucsd.edu

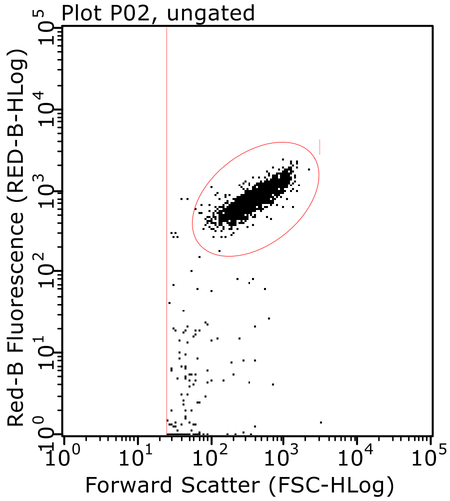

Supplementary Fig. 1. Exemplary plot of flow cytometry gates used to enumerate phytoplankton. Phytoplankton concentrations (cells mL^{-1}) were determined using diagnostic gates of red fluorescence versus forward scatter of exponentially growing monocultures. An exponentially growing population of *M. pusilla* CCMP 1545 is shown.

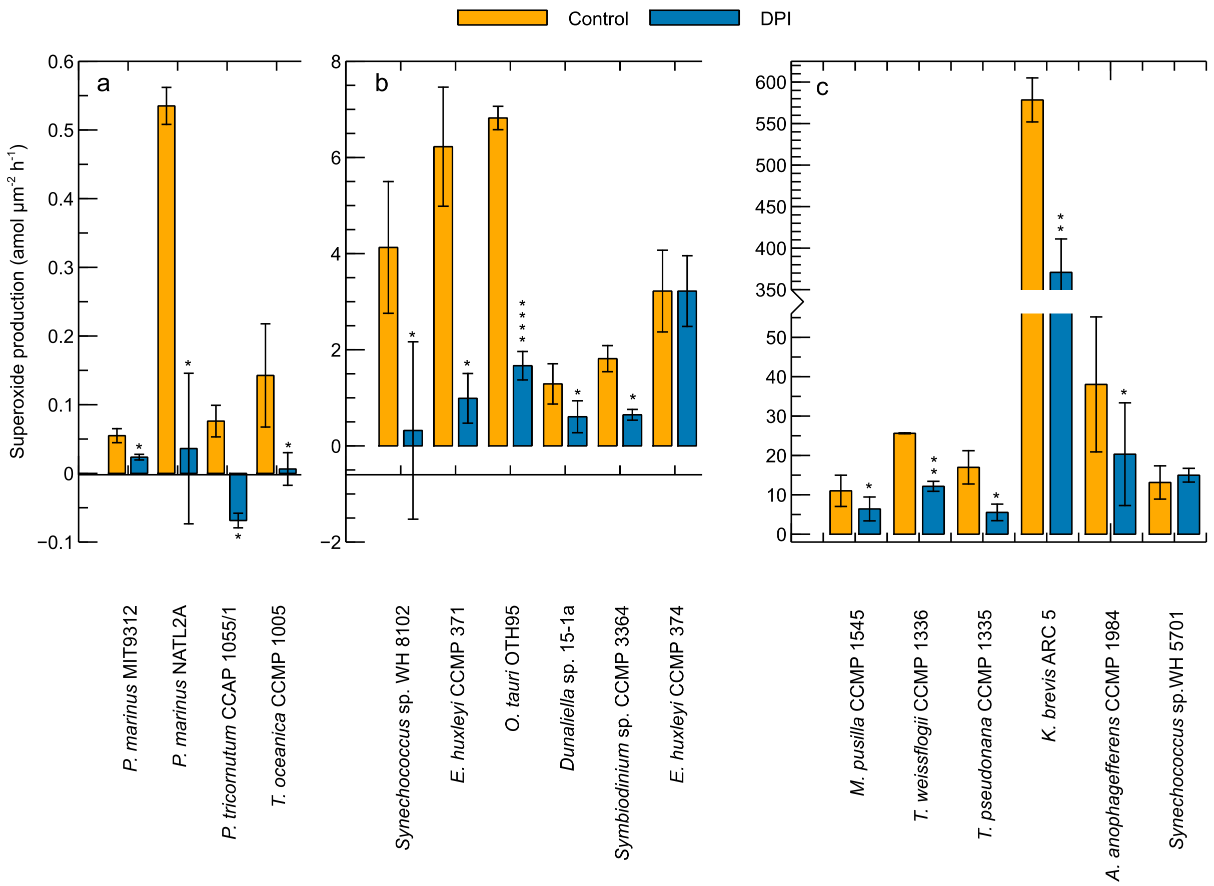

Supplementary Fig. 2. Rates of eO_2^- production in the presence and absence of the flavoenzyme inhibitor DPI. All measurements were conducted in 0.3% DMSO. Production rates were normalized to cell surface area (Supplementary Table 5). Note the different y-axis scales on each panel. Significant differences of mean eO_2^- production rates (n = 3 biological replicates) versus the DMSO control were found with a Student's t-test (paired, two sample). P-values are indicated by asterisks, where *, **, and **** signifies a p-value of <0.05, <0.01, and <0.0001, respectively. Error bars represent one standard deviation of the mean. Data from *T. oceanica* CCMP 1005 were taken from Diaz et al. [1]. Replicate measurements are shown in Supplementary Table 6.

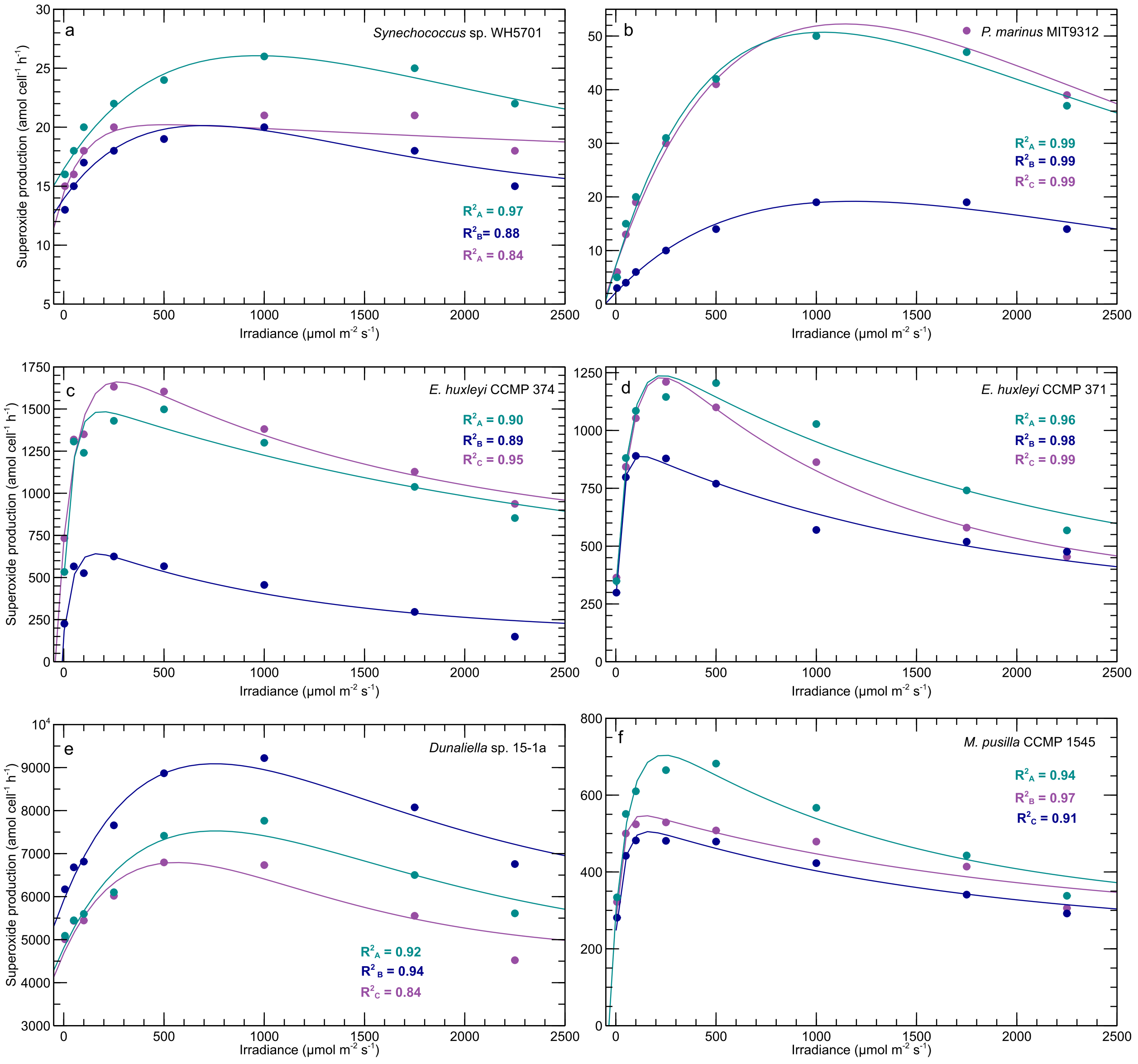

a
Synechococcus sp. WH5701
R^2_A = 0.97
R^2_B= 0.88
R^2_A = 0.84
b
P. marinus MIT9312
R^2_A = 0.99
R^2_B = 0.99
R^2_C = 0.99
c
E. huxleyi CCMP 374
R^2_A = 0.90
R^2_B = 0.89
R^2_C = 0.95
d
E. huxleyi CCMP 371
R^2_A = 0.96
R^2_B = 0.98
R^2_C = 0.99
e
Dunaliella sp. 15-1a
R^2_A = 0.92
R^2_B = 0.94
R^2_C = 0.84
f
M. pusilla CCMP 1545
R^2_A = 0.94
R^2_B = 0.97
R^2_C = 0.91
Superoxide production (amol cell^{-1} h^{-1})
Irradiance (µmol m^{-2} s^{-1})

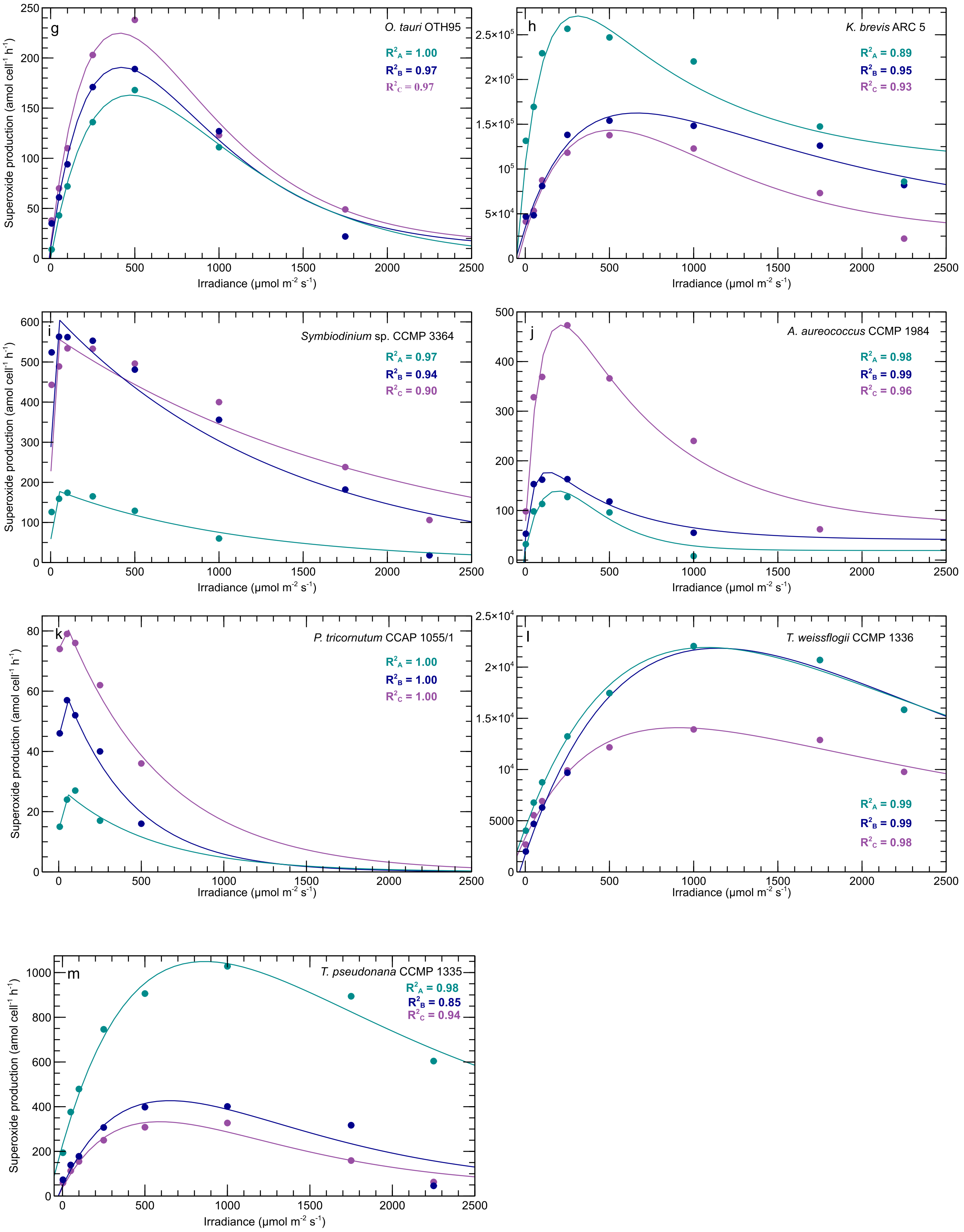

g
O. tauri OTH95
R²A = 1.00
R²B = 0.97
R²C = 0.97
h
K. brevis ARC 5
R²A = 0.89
R²B = 0.95
R²C = 0.93
i
Symbiodinium sp. CCMP 3364
R²A = 0.97
R²B = 0.94
R²C = 0.90
j
A. aureococcus CCMP 1984
R²A = 0.98
R²B = 0.99
R²C = 0.96
k
P. tricornutum CCAP 1055/1
R²A = 1.00
R²B = 1.00
R²C = 1.00
l
T. weissflogii CCMP 1336
R²A = 0.99
R²B = 0.99
R²C = 0.98
m
T. pseudonana CCMP 1335
R²A = 0.98
R²B = 0.85
R²C = 0.94
Superoxide production (amol cell⁻¹ h⁻¹)
Irradiance (µmol m⁻² s⁻¹)

Supplementary Fig. 3. eO_2^- production rates measured at increasing irradiances from triplicate batch cultures of model phytoplankton strains. Irradiance and eO_2^- production rate data (circles) were fit with a photosynthesis-irradiance model by Platt et al. [2] that was adapted by Diaz et al. [1] for eO_2^- production rates (lines). Each color represents a different biological replicate. R^2 values of the model fit for each biological replicate are provided. Model rates are presented in Supplementary Table 2. Results for *T. oceanica* are from Diaz et al. [1] and are not presented here.

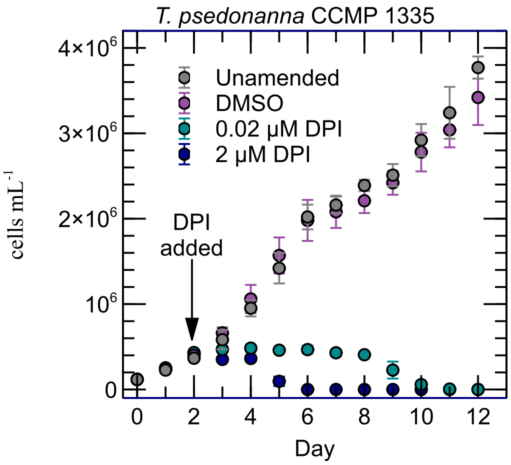

Supplementary Fig. 4. The effect of DPI on growth of *T. pseudonana* CCMP 1335. Error bars show standard deviation of the mean of biological replicates (n=3).

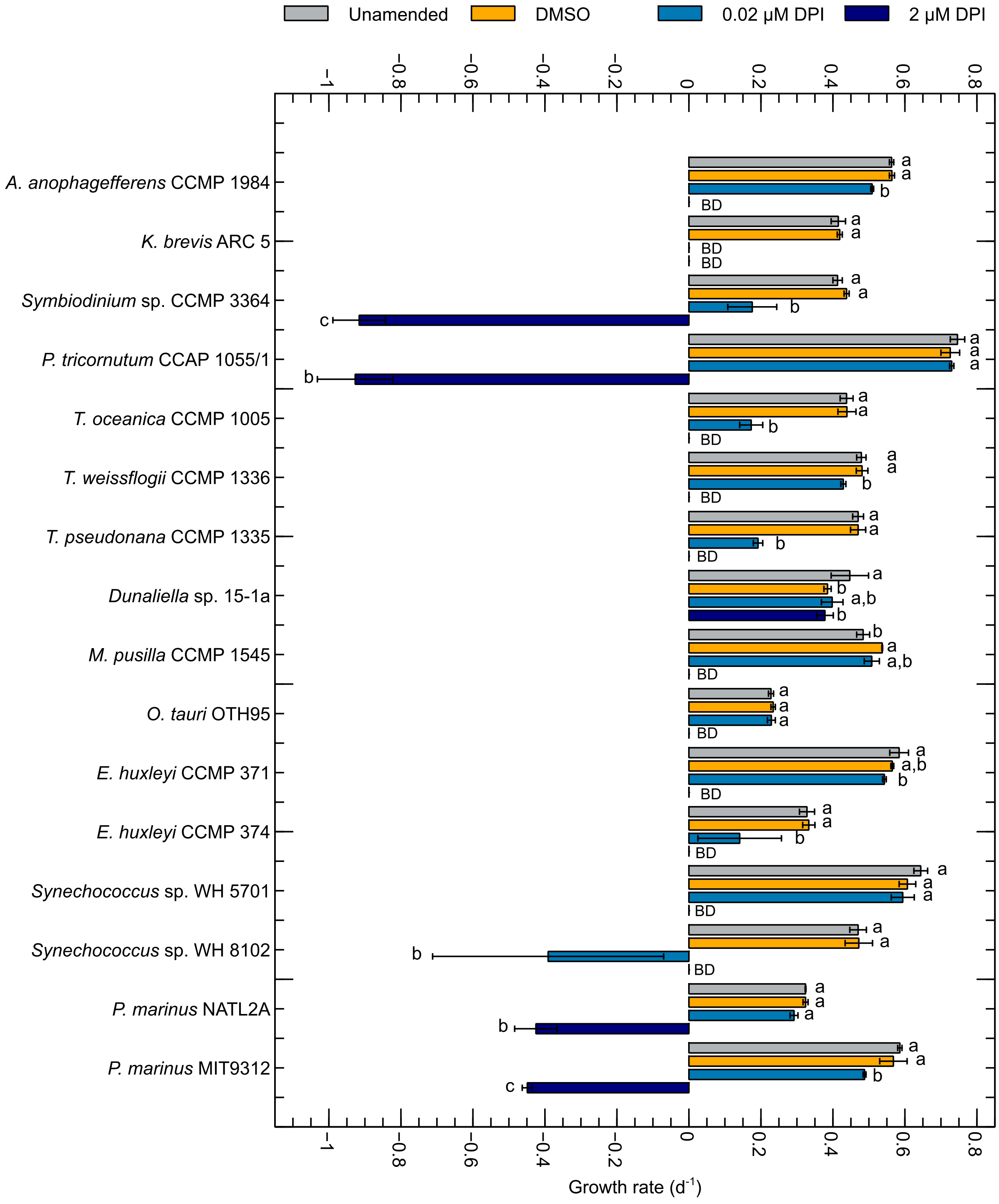

Unamended
DMSO
0.02 µM DPI
2 µM DPI
A. anophagefferens CCMP 1984
K. brevis ARC 5
Symbiodinium sp. CCMP 3364
P. tricornutum CCAP 1055/1
T. oceanica CCMP 1005
T. weissflogii CCMP 1336
T. pseudonana CCMP 1335
Dunaliella sp. 15-1a
M. pusilla CCMP 1545
O. tauri OTH95
E. huxleyi CCMP 371
E. huxleyi CCMP 374
Synechococcus sp. WH 5701
Synechococcus sp. WH 8102
P. marinus NATL2A
P. marinus MIT9312
BD
−1
−0.8
−0.6
−0.4
−0.2
0
0.2
0.4
0.6
0.8
Growth rate (d^{-1})

Supplementary Fig. 5. Specific growth rates in the presence or absence of the flavoenzyme inhibitor DPI. All cultures were grown in 0.03% DMSO, except the unamended control. BD stands for below detection and indicates cell death at a rate that was faster than our limit of detection. Significant differences of mean growth rates > -1.00 (n = 3 biological replicates) between treatments were found using a Student's t-test (unpaired, two-sample). Treatments not connected by the same letter within a strain are significantly different (p-value < 0.05). Error bars show the standard deviation of the mean of biological replicates (n=3). Replicate measurements are shown in Supplementary Table 6.

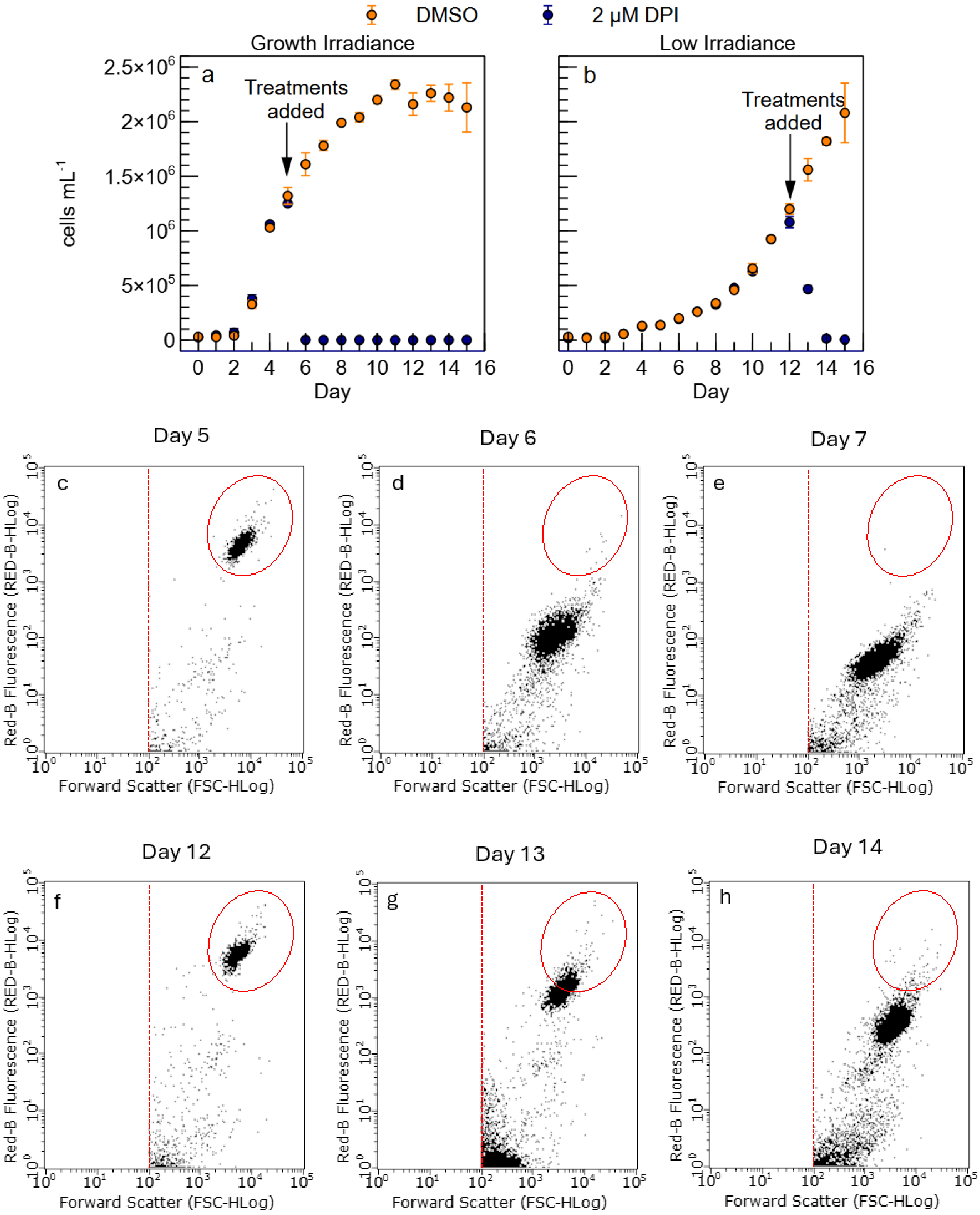

DMSO
2 µM DPI
Growth Irradiance
Low Irradiance
a
b
Treatments added
cells mL-1
Day
Day 5
Day 6
Day 7
Day 12
Day 13
Day 14
c
d
e
f
g
h
Red-B Fluorescence (RED-B-HLog)
Forward Scatter (FSC-HLog)

Supplementary Fig. 6. The effect of DPI on *T. oceanica* CCMP 1005 grown under typical growth irradiance and low irradiance. Growth curves (a,b) show cell concentrations (cells mL^{-1}) over time under ~115 µmol m^{-2} s^{-1} (growth irradiance; a) and ~15 µmol m^{-2} s^{-1} (low irradiance; b) before and after the addition of 2 µM DPI or 0.03% DMSO (arrows). Error bars show standard deviation of the mean of biological replicates (n=3). Flow cytometry plots from the same experiment (c-h) show healthy, exponentially growing cells (red circle) on the day of DPI addition (c,f) and the subsequent decline and death of these cells 24 and 48 hr after DPI addition (d,e,g,h) in cultures grown under growth irradiance (c-e) and low irradiance (f-h).

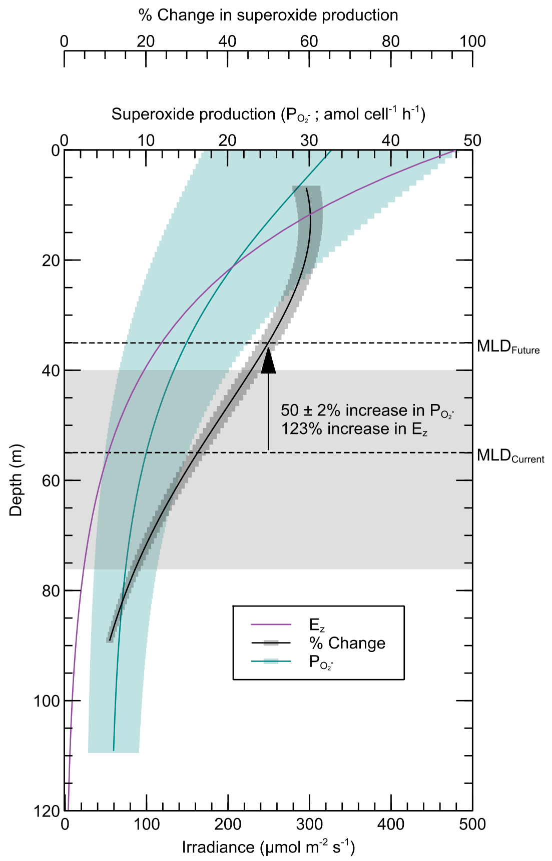

Supplementary Fig. 7. Calculated production rates of eO_2^- by *Prochlorococcus* (P_{O2-}) under current and future conditions in the North Pacific Subtropical Gyre. Production rates of eO_2^- by *Prochlorococcus* (P_{O2-}) and irradiance (E_z) as a function of depth in the North Pacific Subtropical Gyre (Station ALOHA) were based on median surface irradiance or E_0 [3] (see Materials and Methods). Dashed lines indicate median current ($MLD_{Current}$) and future (MLD_{Future}= $MLD_{Current}$ – 20 m) [4] mixed layer depths. Shaded area indicates the 25th and 75th percentile of the $MLD_{Current}$. Black line shows the % change in per-cell eO_2^- production at each depth between the 5th and 95th percentile of the MLD under current and future conditions. Shaded bars around P_{O2-} and % change show standard deviation of the mean of biological replicates (n=3). Using equation (5), we determined that a 20 m shoaling would increase E_z at the MLD by 123% by the year 2200. For instance, a 20 m shoaling from 55 m to 35 m would increase E_z by

~53 μmol m^{-2} s^{-1} to ~119 μmol m^{-2} s^{-1} or 123%. E_z data do not have standard deviation because these data are based on a fundamental equation modeling the relationship between surface irradiance and depth (see Materials and Methods).

Supplementary Table 1. Growth conditions, sources, and ecotypes of the 16 model phytoplankton strains selected for the study. Strains are marine unless otherwise stated. L1 + Si [5], f/2 and f/2 + Si [6], SN [7], and Pro99 [8] media were prepared according to standard procedures.

Class	Genus species	Strain	Media	Growth Irradiance (μmol m^{-2} s^{-1})	Temperature (°C)	Axenic	Ecotype Notes
Prymnesiophyceae	*Emiliania huxleyi*	CCMP 374	f/2	100-130	18	Yes	non-calcifying
	Emiliania huxleyi	CCMP 371	f/2	100-130	18	Yes	calcifying
Chlorophyceae	*Ostreococcus tauri*	OTH95	f/2	100-130	18	No	
	Micromonas pusilla	CCMP 1545	f/2	100-130	18	Yes	
	Dunaliella	15-1a	L1 + Si (120 ppt)	100-130	18	No	hypersaline
Bacillariophyceae	*Thalassiosira pseudonana*	CCMP 1335	f/2 + Si	100-130	18	No	coastal
	Thalassiosira weissflogii	CCMP 1336	f/2 + Si	100-130	18	Yes	coastal
	Thalassiosira oceanica	CCMP 1005	f/2 + Si	100-130	23	Yes	oligotrophic
	Phaeodactylum tricornutum	CCAP 1055/1	L1 + Si	100-130	18	No	
Dinophyceae	*Symbiodinium* sp.	CCMP 3364	L1 + Si	100-130	23	No	coral symbiont
	Karenia brevis	ARC 5	L1 + Si	100-130	23	No	HAB-forming
Pelagophyceae	*Aureococcus anophagefferens*	CCMP 1984	L1 + Si	100-130	18	No	HAB-forming
Cyanophyceae	*Synechococcus* sp.	WH 8102	SN	70-80	23	No	oligotrophic
	Synechococcus sp.	WH 5701	L1 + Si	70-80	23	Yes	coastal
	Prochlorococcus marinus	MIT9312	Pro99	70	23	Yes	high-light adapted
	Prochlorococcus marinus	NATL2A	Pro99	40	23	Yes	low-light adapted

Supplementary Table 2. eO_2^- irradiance curve parameters. eO_2^- production rates and irradiance data for each biological replicate were fit to a photosynthesis-irradiance model modified from [2] by Diaz et al. [1] (see Materials and Methods) * = data from Diaz et al. [1]

Phytoplankton Strain	Biological Replicate	P_D^{O2-}	P_S^{O2-}	P_m^{O2-}	α	β	E_k^{O2-}	R^2
		amol cell^{-1} h^{-1}			$\frac{amol\ cell^{-1}h^{-1}}{\mu mol\ m^{-2}s^{-1}}$		µmol m^{-2} s^{-1}	
Synechococcus sp. WH 8102	A	21	56	68	0.053	0.002	1271	0.97
	B	35	3267	70	0.013	0.457	5222	0.83
	C	28	983	95	0.042	0.206	2261	1.00
Synechococcus sp. WH 5701	A	16	1068	26	0.027	1.092	961	0.97
	B	14	209	20	0.024	0.285	837	0.88
	C	14	6	20	0.049	0.001	414	0.84
Prochlorococcus marinus MIT9312	A	7	101	51	0.122	0.049	414	0.99
	B	2	1864	19	0.039	1.554	492	0.99
	C	7	368	52	0.108	0.272	483	0.99
Prochlorococcus marinus NATL2A	A	37	81	105	0.689	0.028	152	0.92
	B	16	48	50	0.287	0.030	175	0.91
	C	38	126	128	0.738	0.076	173	0.95
Emiliania huxleyi CCMP 374	A	475	1107	1485	23.484	0.431	63	0.90
	B	162	575	641	11.282	0.499	57	0.89
	C	722	1186	1661	12.527	0.764	133	0.95
Emiliania huxleyi CCMP 371	A	298	1099	1238	15.891	0.572	78	0.96
	B	247	704	891	22.836	0.411	39	0.98
	C	315	1194	1230	13.907	1.014	88	0.99
Ostreococcus tauri OTH95	A	1	25286	163	0.930	52.801	175	1.00
	B	10	84543	191	1.169	200.841	163	0.97
	C	14	19863	225	1.401	47.809	160	0.97
Micromonas pusilla CCMP1545	A	295	525	704	6.072	0.402	116	0.94
	B	235	305	505	6.958	0.182	73	0.97
	C	262	311	547	9.163	0.162	60	0.91
Dunaliella sp. 15-1a	A	4807	467192	7527	9.763	612.006	771	0.92
	B	5919	491118	9088	11.450	646.982	794	0.94
	C	4682	429379	6792	10.165	756.058	668	0.84
Symbiodinium sp. CCMP 3364	A	0	186	180	35.457	0.168	5	0.97
	B	0	629	618	190.099	0.458	3	0.94
	C	0	571	563	143.464	0.287	4	0.90
Karenia brevis ARC 5	A	105103	289013	270949	1644.497	342.555	165	0.89
	B	34694	263581	162532	569.618	179.279	285	0.95
	C	28387	88440426	143429	610.110	172241.168	235	0.93
Thalassiosira weissflogii CCMP 1336	A	4298	1063152	21922	44.300	961.037	495	0.99
	B	1699	749719	21841	47.775	630.454	457	0.99
	C	3365	22667	14082	34.611	11.519	407	0.98
Thalassiosira pseudonanna CCMP 1335	A	227	25891	1049	2.590	28.719	405	0.98
	B	44	64341	427	1.599	98.051	267	0.85
	C	37	15096	333	1.364	24.959	244	0.94
Thalassiosira oceanica CCMP 1005*	A	28	33	51	0.390	0.010	131	0.98
	B	17	42	58	0.340	0.002	168	1
	C	29	34	59	0.230	0.007	260	0.99
Phaeodactylum tricornutum CCAP 1055/1	A	0	28	26	3.599	0.051	7	1
	B	0	66	62	13.292	0.159	5	1
	C	0	89	86	27.372	0.147	3	1
Aureococcus anophagefferens CCMP 1984	A	19	9197	139	1.700	47.049	82	0.98
	B	41	205	178	3.358	0.438	53	0.99
	C	68	662	473	5.965	1.028	79	0.96

Supplementary Table 3. Parameters for estimating eO_2^- production rates under current and future conditions of the North Pacific Subtropical Gyre. All data are obtained from the North Pacific gyre or another representative oligotrophic gyre. NPSG = North Pacific Subtropical Gyre, MLD = mixed layer depth

Parameter (units)	Value	Notes	Source
Surface Par or E_0 Range (μmol m^{-2} s^{-1})	324 – 613		Estimated from Figure 2 in Letelier et al. [3]
Surface PAR or E_0 Median (μmol m^{-2} s^{-1})	481		Calculated with data from Letelier et al. [3]
Light attenuation coefficient (*k*; m^{-1})	0.04		Letelier et al.[3]
5th – 95th percentile of the MLD (m)	27 – 109		HOT-DOGS [9]
Median MLD (m)	55		HOT-DOGS [9]
Prochlorococcus Abundance (cells L^{-1})	5.7×10^7	Divided 10×10^{12} m^{-2} by listed depth integration (175 m)	Table 2 in Bjorkman et al. [10]
Synechococcus Abundance Range (cells L^{-1})	4.17×10^5 – 2.5×10^6	Divided 0.5×10^{11} m^{-2} by shallowest (20 m) and deepest MLD (120 m)	HOT-DOGS [9]
Diatoms Abundance (cells L^{-1})	8.33×10^2 – 5×10^3	Divided 1×10^8 m^{-2} by shallowest (20 m) and deepest MLD (120 m)	Estimated from Figure 1 in Scharek et al. [11]
Ostreococcus Abundance (cells L^{-1})	10^3		Figure 9 from Not et al. [12]
Micromonas Abundance (cells L^{-1})	7×10^3 – 21×10^3		Table VI in Furuya et al. [13]
Coccolithophore Abundance (cells L^{-1})	10^3		Figure 3 in Cortés et al. [14]

Supplementary Table 4. Current and future ocean conditions estimated for the North Pacific Subtropical Gyre. MLD = mixed layer depth, E_0 = surface irradiance or irradiance at 0 m, E_z = irradiance at depth z, $P^{O_2^-}_{in\ situ}$ = production of eO_2^- by *Prochlorococcus*

		E_0 = 324 µmol m^{-2} s^{-1}			E_0 = 481 µmol m^{-2} s^{-1}			E_0 = 613 µmol m^{-2} s^{-1}		
**Ocean Conditions**	**MLD (m)**	**E_z (µmol m^{-2} s^{-1})**	**$P^{O_2^-}_{in\ situ}$ (nM d^{-1})**	**$P^{O_2^-}_{in\ situ}$ (amol cell^{-1} h^{-1})**	**E_z (µmol m^{-2} s^{-1})**	**$P^{O_2^-}_{in\ situ}$ (nM d^{-1})**	**$P^{O_2^-}_{in\ situ}$ (amol cell^{-1} h^{-1})**	**E_z (µmol m^{-2} s^{-1})**	**$P^{O_2^-}_{in\ situ}$ (nM d^{-1})**	**$P^{O_2^-}_{in\ situ}$ (amol cell^{-1} h^{-1})**
Future	7	244.9	31 ± 15	23 ± 11	363.5	39 ± 19	29 ± 14	463.3	44 ± 21	32 ± 15
Current	27	110.0	20 ± 10	14 ± 7	163.3	25 ± 12	18 ± 9	208.2	28 ± 14	21 ± 10
Future	35	79.9	17 ± 8	12 ± 6	118.6	21 ±10	15 ± 7	151.2	24 ± 12	17 ± 9
Current	55	53.3	12 ± 6	9 ± 4	53.3	14 ± 7	10 ± 5	67.9	15 ± 8	11 ± 6
Future	89	9.2	9 ± 4	6 ± 3	13.7	9 ± 5	7 ± 3	17.4	10 ± 5	7 ± 4
Current	109	4.1	8 ± 4	6 ± 3	6.1	8 ± 4	6 ± 3	7.8	8 ± 4	6 ± 3

Supplementary Table 5. Cell size and morphology data for surface area calculations of phytoplankton strains. Radius was calculated as $\frac{width}{2}$. For prolate spheroids surface area calculations, $A = \frac{length}{2}$, $B = \frac{width}{2}$, and $m = \sqrt{1 - (\frac{B}{A})^2}$. NCMA = National Center for Marine Algae and Microbiota

Genus species	Strain	Length (μm)	Width (μm)	Radius (μm)	Shape	Surface Area Formula	Surface Area (μm²)	Reference
Emiliania huxleyi	CCMP 374	6	5	2.5	sphere	$4\pi r^2$	78.5	NCMA
Emiliania huxleyi	CCMP 371	7	7	3.5	sphere	$4\pi r^2$	153.9	NCMA
Ostreococcus tauri	OTH95	1	0.7	0.35	sphere	$4\pi r^2$	3.1	Courties et al. [15]
Micromonas pusilla	CCMP 1545	3	2.5	-	prolate spheroid	$2\pi B^2(1 + \frac{Asin^{-1}(m)}{mB})$	22.3	NCMA
Dunaliella	15-1a	8.3	3.3	-	prolate spheroid	$2\pi B^2(1 + \frac{Asin^{-1}(m)}{mB})$	71.6	Microscopy
Thalassiosira pseudonana	CCMP 1335	-	-	-	cylinder	$2\pi rh + 2\pi r^2$	51.0	Kustka et al. [16]
Thalassiosira weissflogii	CCMP 1336	-	-	-	cylinder	$2\pi rh + 2\pi r^2$	460.0	Kustka et al. [16]
Thalassiosira oceanica	CCMP 1005	-	-	-	cylinder	$2\pi rh + 2\pi r^2$	122.0	Lommer et al. [17]
Phaeodactylum tricornutum	CCAP 1055/1	22	2.5	-	pennate	-	368.0	Leblanc et al [18]
Symbiodinium sp.	CCMP 3364	13.5	11.5	-	prolate spheroid	$2\pi B^2(1 + \frac{Asin^{-1}(m)}{mB})$	464.4	Zhang et al. [19]
Karenia brevis	ARC 5	25	28	14	sphere	$4\pi r^2$	2461.8	Novoveská et al. [20]
Aureococcus anophagefferens	CCMP 1984	-	-	1.1	sphere	$4\pi r^2$	15.2	Sieburth and Johnson [21]
Synechococcus sp.	WH 8102	1.5	1.5	0.75	sphere	$4\pi r^2$	7.1	NCMA
Synechococcus sp.	WH 5701	1	1	0.5	sphere	$4\pi r^2$	3.1	NCMA
Prochlorococcus marinus	MIT9312	1.4	0.7	-	prolate spheroid	$2\pi B^2(1 + \frac{Asin^{-1}(m)}{mB})$	2.6	NCMA
Prochlorococcus marinus	NATL2A	1.4	0.7	-	prolate spheroid	$2\pi B^2(1 + \frac{Asin^{-1}(m)}{mB})$	2.6	NCMA

Supplementary References

1. Diaz JM, Plummer S, Hansel CM, Andeer PF, Saito MA, McIlvin MR. NADPH-dependent extracellular superoxide production is vital to photophysiology in the marine diatom *Thalassiosira oceanica*. *Proc Natl Acad Sci U S A* 2019;**116**:16448-53. https://doi.org/10.1073/pnas.1821233116
2. Platt T, Gallegos C.L., Harrison W.G. Photoinhibition of photosynthesis in natural assemblages of marine phytoplankton. *J Mar Res* 1980;**38**:687-701.
3. Letelier RM, Karl DM, Abbott MR, Bidigare RR. Light driven seasonal patterns of chlorophyll and nitrate in the lower euphotic zone of the North Pacific Subtropical Gyre. *Limnol Oceanogr* 2004;**49**:508-19.
4. Luo Y, Rothstein LM. Response of the Pacific Ocean circulation to climate change. *Atmos-Ocean* 2011;**49**:235-44. https://doi.org/10.1080/07055900.2011.602325
5. Guillard RRL, Hargraves PE. *Stichochrysis immobilis* is a diatom, not a chrysophyte. *Phycologia* 1993;**32**:234-36. https://doi.org/10.2216/i0031-8884-32-3-234.1
6. Guillard RRL, Ryther JH. Studies of marine planktonic diatoms: I. *Cyclotella nana hustedt*, and *Detonula confervacea (cleve) gran*. *Can J Microbiol* 1962;**8**:229-39. https://doi.org/10.1139/m62-029
7. Waterbury JB. Biological and ecological characterization of the marine unicellular Cyanobacterium *Synechococcus*. *Photosynthetic picoplankton Canadian Bulletin of Fisheries Aquatic Sciences* 1987;**214**:71-120.
8. Moore LR, Coe A, Zinser ER, Saito MA, Sullivan MB, Lindell D *et al.* Culturing the marine cyanobacterium *Prochlorococcus*. *Limnol Oceanogr: Methods* 2007;**5**:353-62.
9. Data obtained via the Hawaii Ocean Time-series HOT-DOGS application; University of Hawai'i at Mānoa. National Science Foundation Award # 1756517.
10. Björkman KM, Church MJ, Doggett JK, Karl DM. Differential assimilation of inorganic carbon and leucine by *Prochlorococcus* in the oligotrophic North Pacific Subtropical Gyre. *Front Microbiol* 2015;**6**:1401.
11. Scharek R, Latasa M, Karl DM, Bidigare RR. Temporal variations in diatom abundance and downward vertical flux in the oligotrophic North Pacific gyre. *Deep Sea Res I: Oceanogr Res Pap* 1999;**46**:1051-75.
12. Not F, Latasa M, Scharek R, Viprey M, Karleskind P, Balagué V *et al.* Protistan assemblages across the Indian Ocean, with a specific emphasis on the picoeukaryotes. *Deep Sea Res I: Oceanogr Res Pap* 2008;**55**:1456-73.
13. Furuya K, Marumo R. The structure of the phytoplankton community in the subsurface chlorophyll maxima in the western North Pacific Ocean. *J Plankton Res* 1983;**5**:393-406.
14. Cortés MY, Bollmann J, Thierstein HR. Coccolithophore ecology at the HOT station ALOHA, Hawaii. *Deep Sea Res II: Top Stud Oceanogr* 2001;**48**:1957-81.
15. Courties C, Vaquer A, Troussellier M, Lautier J, Chretiennotdinet M, Neveux J *et al.* Smallest eukaryotic organism. *Nature* 1994;**370**:255.
16. Kustka AB, Shaked Y, Milligan AJ, King DW, Morel FMM. Extracellular production of superoxide by marine diatoms: Contrasting effects on iron redox chemistry and bioavailability. *Limnol Oceanogr* 2005;**50**:1172-80. https://doi.org/DOI 10.4319/lo.2005.50.4.1172
17. Lommer M, Specht M, Roy A-S, Kraemer L, Andreson R, Gutowska MA *et al.* Genome and low-iron response of an oceanic diatom adapted to chronic iron limitation. *Genome Biol* 2012;**13**:1-21.
18. Leblanc K, Arístegui Ruiz J, Armand L, Assmy P, Beker B, Bode A *et al.* Global distributions of diatoms abundance, biovolume and biomass-Gridded data product (NetCDF)-Contribution to the MAREDAT World Ocean Atlas of plankton functional types. *Dataset PANGAEA https://doi org/101594/PANGAEA* 2012;**777384**

19. Zhang T, Diaz JM, Brighi C, Parsons RJ, McNally S, Apprill A *et al.* Dark production of extracellular superoxide by the coral *Porites astreoides* and representative symbionts. *Front Mar Sci* 2016;**3** https://doi.org/10.3389/fmars.2016.00232
20. Novoveská L, Robertson A. Brevetoxin-producing spherical cells present in *Karenia brevis* bloom: evidence of morphological plasticity? *J Mar Sci Eng* 2019;**7**:24.
21. Sieburth JM, Johnson PW Picoplankton ultrastructure: a decade of preparation for the brown tide alga, *Aureococcus anophagefferens*. In: *In: Cosper, E.M., Bricelj, V.M., Carpenter, E.J. (eds) Novel Phytoplankton Blooms. Coastal and Estuarine Studies*, Berlin, Heidelberg: Springer. 1-21.
